# A Systematic Review on Dementia and Translocator Protein (TSPO): When Nuclear Medicine Highlights an Underlying Expression

**DOI:** 10.3390/biom13040598

**Published:** 2023-03-26

**Authors:** Miriam Conte, Maria Silvia De Feo, Ferdinando Corica, Joana Gorica, Marko Magdi Abdou Sidrak, Flaminia De Cristofaro, Luca Filippi, Maria Ricci, Giuseppe De Vincentis, Viviana Frantellizzi

**Affiliations:** 1 Department of Radiological Sciences, Oncology and Anatomo-Pathology, Sapienza, University of Rome, 00161 Rome, Italy; 2 Department of Nuclear Medicine, Santa Maria Goretti Hospital, 04100 Latina, Italy; 3 Nuclear Medicine Unit, Cardarelli Hospital, 86100 Campobasso, Italy

**Keywords:** TSPO, PBR, neuroinflammation, SPECT, PET, dementia

## Abstract

Background: Translocator protein (TSPO) is a neuroinflammation hallmark. Different TSPO affinity compounds have been produced and over time, the techniques of radiolabeling have been refined. The aim of this systematic review is to summarize the development of new radiotracers for dementia and neuroinflammation imaging. Methods: An online search of the literature was conducted in the PubMed, Scopus, Medline, Cochrane Library, and Web of Science databases, selecting published studies from January 2004 to December 2022. The accepted studies considered the synthesis of TSPO tracers for nuclear medicine imaging in dementia and neuroinflammation. Results: A total of 50 articles was identified. Twelve papers were selected from the included studies’ bibliographies and 34 were excluded. Thus, 28 articles were ultimately selected for quality assessment. Conclusion: Huge efforts in developing specific and stable tracers for PET/SPECT imaging have been made. The long half-life of ^18^F makes this isotope a preferable choice to ^11^C. An emerging limitation to this however is that neuroinflammation involves all of the brain which inhibits the possibility of detecting a slight inflammation status change in patients. A partial solution to this is using the cerebellum as a reference region and developing higher TSPO affinity tracers. Moreover, it is necessary to consider the presence of distomers and racemic compounds interfering with pharmacological tracers’ effects and increasing the noise ratio in images.

## 1. Introduction

Translocator protein (TSPO), previously designated as peripheral benzodiazepine receptor (PBR), is a protein on the outer mitochondrial membrane with five transmembrane helical domains combined with a voltage-dependent anion channel and a nucleoside carrier. It has been well conserved throughout evolution and is expressed both by prokaryotes and eukaryotes. TSPO binds benzodiazepines, namely RO5-4864 and derivates of isoquinoline carboxamide including PK11195 [1]. In fact, this protein belongs to the family of benzodiazepine receptors which can be classically distinguished into two types of receptors: the central benzodiazepine receptor (CBR) localized in the central nervous system, through which benzodiazepines exert their pharmacological effects [2], and the peripheral benzodiazepine receptor (PBR), or peripheral benzodiazepine binding site (PBBS) (Schoemaker et al. 1981), the object of this systematic review. TSPO can be found in steroid cells as well as in the central nervous system. It is ubiquitous in cerebral phagocytic cells (microglia), which are the primary immune cells in the central nervous system, but it can also be found in the heart, lungs, kidneys, and liver as well [3,4,5,6]. The primary function of TSPO is its involvement in cholesterol transportation through the inner mitochondrial membrane for steroid synthesis. TSPO seems to be involved in the regulation of mitochondrial membrane potential and metabolism, apoptosis and proliferation, inflammation, immunomodulation, the transport of porphyrin, heme synthesis, calcium signaling, and in the regulation of oxidative stress [1]. Regarding its regulation, in a normal brain, density expression is modest [7]. On the contrary, TSPO expression is upregulated in activated microglia, which occurs after an injury, in the choroid plexus and, though in lower amounts, in reactive astrocytes and the olfactory bulb’s neurons. Upregulation can also be observed in two brain malignancies: neuroblastoma and glioblastoma [8]. TSPO is therefore considered a sensitive hallmark for microglial activation and glial response. Since inflammation is tightly linked to neuronal dysfunction and loss in Alzheimer’s disease (AD), TSPO could represent a promising tool for neuroinflammation detection and in particular, in conditions such as AD [9]. In fact, inflammation seems to be a phenomenon that predates amyloid deposition while the microglial response holds off Aβ plaque formation [10]. As glial function lowers with aging, microglia become more sensitive to stimuli and less efficient in removing damaged cells which themselves are a potent inflammatory trigger. This phenomenon is a vicious circle that leads to ulterior neuronal damage [11]. A variety of radiolabeled ligands has been produced for PET and SPECT imaging [12,13]. Their low affinity, low binding specificity, and high lipophilicity which is reflected in high background noise [14] have led to the development of novel second- and third-generation radiotracers. To name a few, the most famous TSPO PET tracer is ^11^C-PK11195, while the newest tracers are ^11^C-SSR180575, ^11^C-PBR28, ^18^F-FEDAA1106, ^18^F-PBR06, ^18^F-FEPPA, ^11^C-DPA713, ^18^F-PBR111, ^11^C-DAC, ^11^C-AC-5216, ^18^F-DPA-714, and ^123^I-CLINDE [15]. However, newer generation tracers are affected by variability in binding affinity due to human genetic polymorphism (rs6971) in exon 4 of the TSPO gene, which determines the substitution of alanine for threonine at position 147 (A147T) [16]. To give an example, Owen et al. discovered that ^11^C-PBR28 did not bind in a cohort of non-binding patients; however, this was not detected using ^11^C-PK11195 [17]. Based on this premise, tracers can be divided into high-affinity binders (HABs), low-affinity binders (LABs), and mixed-affinity binders (MABs). The predominant polymorphism Ala/Ala is responsible for high affinity, while the forms Ala/Thr and Thr/Thr are involved in mixed and low affinity, respectively [15]. Since in low-affinity binders, the quality of the image is degraded, a feasible genetic leukocyte analysis could be conducted to determine the TSPO polymorphism. Difficulties in establishing a reference region remain in patients with huge widespread inflammation, in which a slight neuroinflammation status change could be challenging to detect. A partial solution has been found using the cerebellum as a reference region for ^11^C-PBR28-TSPO binding in AD even if further studies for the other tracers are needed [18]. In addition, a pivotal aspect to contemplate in pharmacology is the presence of enantiomers of a considered molecule and thus the racemic form of a mixture. For instance, GE180 is a chiral molecule synthesized as a racemate and its S-enantiomer has the highest affinity for TSPO and better brain uptake specificity and good clearance than the R- one [19]. Enantiomers have different pharmacokinetic and pharmacodynamic properties compared to the racemic form and distomers (less active enantiomers) can be present. Racemic drugs have differences between each other since they can have a single bioactive enantiomer or equally active enantiomers or, again, chiral inversion. All these features influence the safety, toxicity, and efficacy of a molecule. Thus, it is vital to separate the different enantiomers and to radiolabel the useful and effective ones [20,21]. For example, GE180 vibrational circular dichroism spectroscopy was used to separate S- and R-isomers in a study by Freedman et al. in 2003 [22]. This systematic review aims to summarize the novel available radiopharmaceuticals used for TSPO detection and explore the steps forward that scientific research has made in TSPO detection in dementia and neuroinflammation. 

## 2. Materials and Methods

### 2.1. Search Strategy and Study Selection

This systematic review was written in line with PRISMA guidelines [23]. An online search of the literature was conducted in the PubMed, Scopus, Medline, Central (Cochrane Library), and Web Of Science databases. Papers published from January 2004 to December 2022 were searched. The following keywords were used in each database: “radiolabeled” AND “TSPO” AND “brain”. Eligible studies had considered the radiopharmaceutical synthesis of radiolabeled TSPO to develop new radiopharmaceuticals for nuclear medicine imaging in dementia and neuroinflammation. Reviews, studies not related to dementia, studies on brain injury, and studies not related to the subject of the research were excluded. Studies being written in the English language was mandatory.

### 2.2. Quality of the Selected Studies

General data such as the author(s), journal, year of publication, country, and study design were retrieved for each article. The selected studies were analyzed through the Quality Assessment of Diagnostic Accuracy Studies-2 (QUADAS2) tool. Data extraction and quality assessment were separately conducted by two reviewers. Any disagreements were resolved through discussion among researchers.

## 3. Results

### 3.1. Search Results

**Figure 1:**
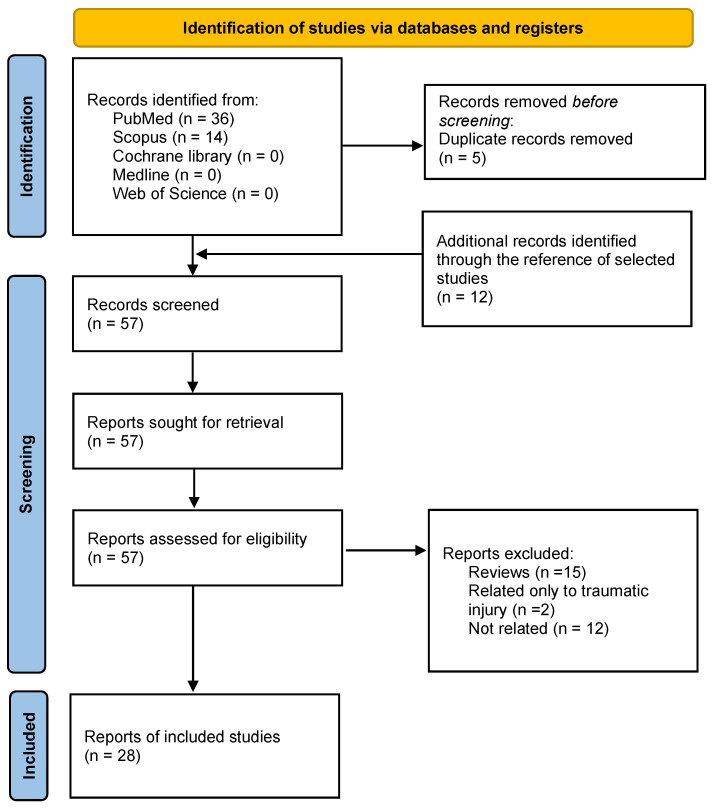
PRISMA workflow for the selection of articles.

The research produced a total of 50 articles. A total of 36 articles was identified from PubMed and 14 from Scopus, while no studies were detected in the Cochrane Library, Medline, or Web of Science. Five duplicate records were removed. The references of the selected studies were examined to check for any additional relevant articles and 12 papers were identified. A total of 57 articles was screened by examining each abstract to identify studies with potential relevance. From the overall group of 57, 29 articles were excluded because they did not satisfy the inclusion criteria. Among them, 15 were reviews, two articles were related only to traumatic injury and 12 studies were not related. The remaining 28 articles were included and selected for quality assessment. The search strategy and selection criteria applied are represented in a flowchart (see Figure 1).

### 3.2. Study Characteristics

The main thematic areas of the selected studies can be summarized as follows: (1) studies of new radiopharmaceuticals with an animal model; (2) biodistribution studies conducted on animals and humans; and (3) studies performed only on humans. The majority of the selected articles were conducted by European researchers, followed by Asian, American, and Australian. Among the selected studies, 15 experiments on tracer biodistribution were performed on animals, six both on humans and animals, and seven on humans only.

### 3.3. Methodological Quality Assessment

**Figure 2:**
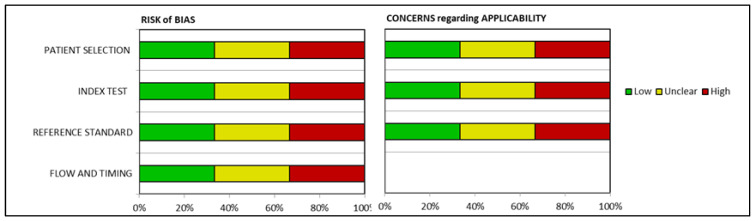
Bias risk results assessed through QUADAS-2.

The methodological quality of the papers included studies of a very high quality. Of the 28 selected studies, only two did not satisfy all the QUADAS-2 domains (see Table 1). From analyzing the results within each bias assessment domain independently (see Table 2), almost all studies obtained a low concern of bias and no more than one study showed high risk in one domain. Considering all four bias assessment domains, three studies reported unclear results, while one study had high-risk results. Regarding patient selection, only two studies reported unclear results, inflow, and timing: one study had unclear results, while in the references and standards used, only one study was high-risk, due to an insufficient number of details. A low concern of applicability of was found for all the studies (See Figure 2).

## 4. Discussion

### 4.1. Studies of New Radiopharmaceuticals Using an Animal Model

In 2004, TSPO “cold” ligands were developed by Okubo et al. from FIGN-1-27 (compound **3**) [47]. Their binding affinity was proven on crude mitochondrial fractions derived from a rat cerebral cortex using a competitive assay with [3H]PK11195. The compound **12a** [(±)-6,11-Dihydro-5-thia-11-aza-benzo[a]fluorene-6-carboxylic acid dihexylamide] had a high affinity for the translocator protein with an IC_50_ = 3.8 nM, while compounds **12e** [(±)-6,11-Dihydro-5-thia-11-aza-benzo [a]fluorene-6-carboxylic acid diethylamide] and **12f** [(±)-6,11-Dihydro-5-thia-11-aza-benzo[a]fluorene-6-carboxylic acid dipropylamide] had values of 0.44 and 0.97 nM for IC_50_, respectively, resulting in being deemed more efficient ligands. The compounds **34a** [(±)-5,6-Dihydrobenzo [4,5]imidazo[2,1-a]isoquinoline-6-carboxylic acid dihexylamide] and 34c [ (±)-5,6-Dihydrobenzo[4,5]imidazo[2,1-a]isoquinoline-6-carboxylic acid dipropylamide], meanwhile, exhibited a moderate affinity for TSPO with their IC_50_ equal to 42 and 74 nM, respectively.

**Figure 3:**
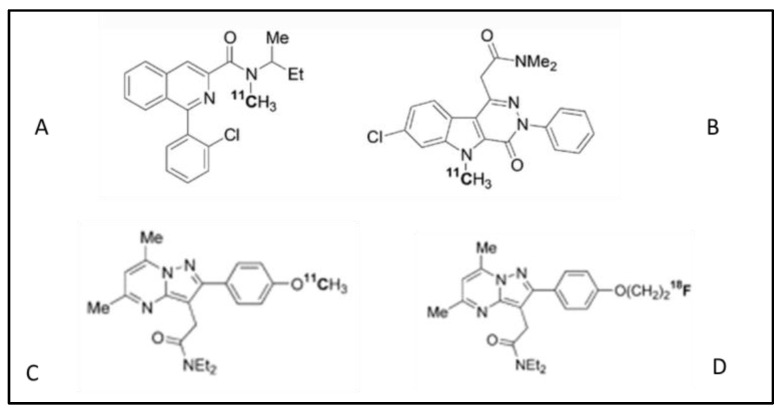
(**A**) ^11^C-PK11195 (or [^11^C]1), a component of the isoquinoline family; (**B**) indoleacetamide-derived 7-chloro-N,N,5-trimethyl-4-oxo-3-phenyl-3,5-dihydro-4H-pyridazino [4,5-b]indole-1-acetamide labeled with ^11^C; (**C**) ^11^C-labeled N,N-diethyl-2- [2-(4-methoxyphenyl)-5,7-dimethylpyrazolo [1,5-a]pyrimidin-3-yl] acetamide [**^11^C-DPA-713**]; (**D**) ^18^F-labeled N,N-diethyl-2-(2-(4-(2-fluoroethoxy)phenyl)-5,7-dimethylpyrazolo [1,5-a]pyrimidin-3-yl) acetamide (^18^F-DPA-714 or ^18^F [2]). DPA-713 and DPA-714 belong to the pyrazolopyrimidine acetamide family.

Chauveau et al. radiolabeled SSR180575 with ^11^C (Figure 3) and performed in vivo and in vitro imaging using a model of rodent acute neuroinflammation comparing with [^11^C](R)-PK11195 [24]. They injected 0.5 μL of (R,S)-α-amino-3-hydroxy-5-methyl-4-isoxazolepropionic (AMPA) into the right striatum of anaesthetized rats. Higher uptake of [^11^C]SSR180575 in the AMPA-lesioned striatum was observed. In the right striatum, the uptake was higher for [^11^C]SSR18057 than [^11^C](R)-PK11195 while the uptake in the contralateral striatum was lower for [^11^C]SSR18057 than [^11^C](R)-PK11195. This result demonstrated that [^11^C]SSR18057 had a better capacity for the discrimination of healthy tissue compared to [^11^C](R)-PK11195.

Another study by Chauveau et al. [25], compared ^11^C-labeled N,N-diethyl-2- [2-(4-methoxyphenyl)-5,7-dimethylpyrazolo [1,5-a]pyrimidin-3-yl] acetamide (^11^C-DPA-713) and ^18^F-labeled N,N-diethyl-2-(2-(4-(2-fluoroethoxy)phenyl)-5,7-dimethylpyrazolo [1,5-a]pyrimidin-3-yl) acetamide (^18^F-DPA-714), two molecules that belong to the pyrazolopyrimidine class (see Figure 3). The study was conducted in vivo and in vitro with a murine model of neuroinflammation. In vitro, ^11^C-DPA-713 and ^18^F-DPA-714 had a similar capability in the detection of neuroinflammatory areas and a similar signal-to-noise ratio. In vivo, the fluorinated compound had the highest ratio of ipsilateral to contralateral uptake and a grated greater binding affinity compared to ^11^C-DPA-713 and ^11^C-PK11195.

**Figure 4:**
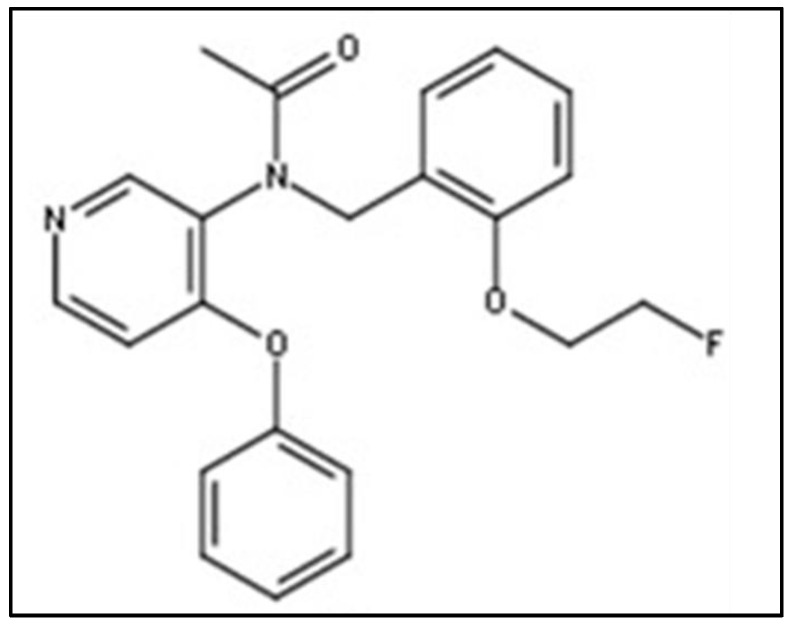
Chemical structure of (2-(2-((*N*-4-phenoxypyridin-3-yl)acetamido)methyl)phenoxy)ethyl 4-methylbenzenesulfonate) radiolabeled with F-18 ([^18^F]FEPPA) which belongs to the family of phenoxypyridinylacetamides.

Another intriguing study is the research of Vignal et al. [26]. They applied the optimized [^18^F]FEPPA (see Figure 4) in a mouse model of cerebral inflammation elicited by the intraperitoneal injection of Salmonella enterica serovar typhimurium lipopolysaccharides preceding a 24 h PET scan. The tracer was synthesized through nucleophilic substitution with a tosylated precursor. The tracer distribution was dependent on TSPO mouse expression as well as in the heart and kidneys, as observed during small animal PET acquisition. Western blotting showed a 2.2-fold greater expression of TSPO in the brain of treated mice than in the control.

**Figure 5:**
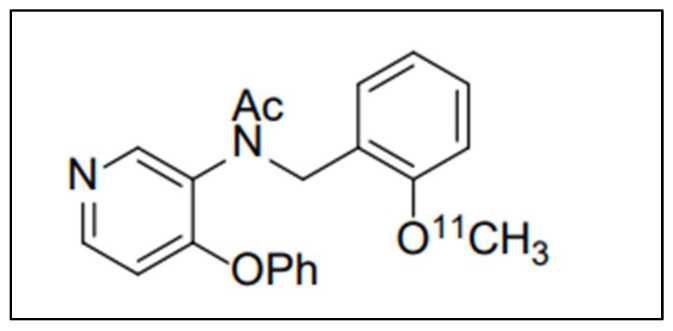
Chemical structure of the aryloxyanilide-based tracer N-(2-[ 11C]methoxybenzyl)-N-(4-phenoxypyridin-3-yl)acetamide, ([11C]PBR28) belonging to the phenoxypyridinylacetamides family.

Solingapuram et al. undertook a biodistribution study of [^11^C]PBR28 (Figure 5) in the male rhesus monkey [27]. A dynamic PET scan of 100 min was executed. After 10 min, cerebellum and basal ganglia uptake were evaluated with a complete washout in 2 h.

**Figure 6:**
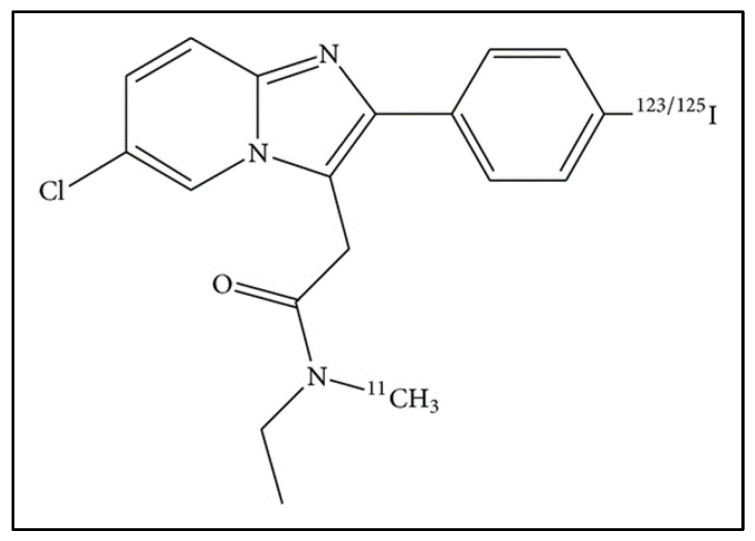
Chemical structure of 6-chloro-2-(4′-iodophenyl)-3-(N,N-methylethyl)imidazo[1,2-a]pyridine-3-acetamide (CLINME) radiolabeled with iodine-123, a compound of the 2-arylimidazo[1,2-a]pyridine-3-acetamide family.

Another fascinating tracer with possible applications in SPECT imaging is [^123^I]-CLINME. Mattener et al. studied the biodistribution of [^123^I]-CLINME (Figure 6) in an AMPA-induced excitotoxic murine model through SPECT imaging [28]. After 30 min to 6 h post-injection, a plateau in the adrenal glands was seen. Maximal activity was reached in the heart, lungs, liver, spleen, and kidneys at 5–15 min post tracer administration, while blood concentration was very low during the exam. Higher activity was detected in the olfactory bulbs compared to the rest of the brain. Thyroid uptake rose from 5 min to 1 h post-injection, then diminished at 3 h to 4%, remaining steady until 6 h post-injection. Competition studies conducted of PK11195 and Ro 5-4854 confirmed the specificity of CLINME binding to TSPO linking sites.

Tran et al. tested a variety of TSPO ligands (11a–c and 13a–d) with a 2-phenylpyrazolo[1,5-a]pyrimidin-3-yl acetamide structure by conducting an in vitro binding assay [29]. A major part of the compound had a better affinity than DPA-714, with particular affinity and lipophilicity in compound **11a**. It was radiolabeled with ^18^F and PET imaging was conducted in an LPS-induced neuroinflammatory murine model. [^18^F]11a demonstrated a high affinity for inflammation sites, confirmed by an immunohistochemical exam conducted on the dissected brain, which showed how the PET uptake was in correspondence with areas of activated microglia.

Qiao et al. conducted a synthesis study on compound **6** (GE387) radiolabeled with ^18^F [16]. The racemic [^18^F]6 was studied in male Wistar rats: the tracer showed low sensitivity to the polymorphism rs6971 in human embryonic kidney cell lines and modest affinity in murine brains because the animals were healthy.

**Figure 7:**
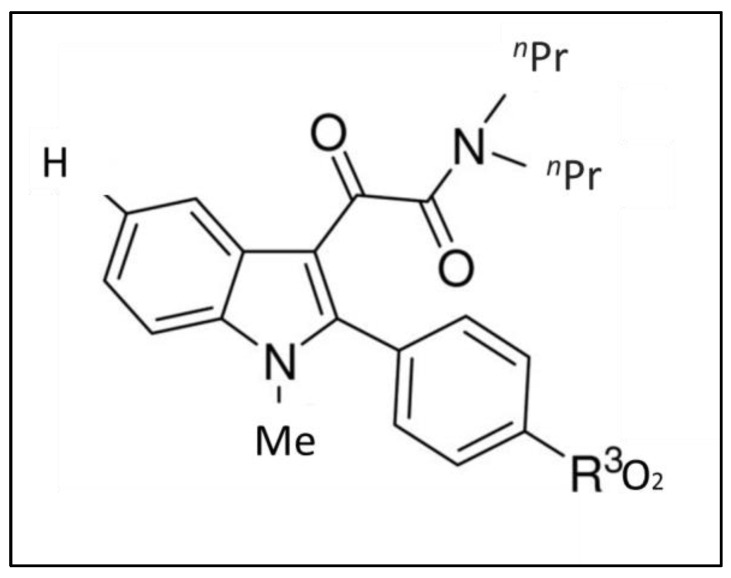
Chemical structure of N1-methylated derivative N,N-di-n-propyl-(N1-methyl-2-(4’-nitrophenyl)indol-3-yl)glyoxylamide (ligand 31).

Pike et al. synthesized a library of new 2-phenylindol-3-ylglyoxylamide derivatives of the general formula II (compounds **19**–**31**), attributing a methyl group on the indole nitrogen. Then, they tested ligand 31 (Figure 7) which, thanks to its methyl group, was feasible to label with carbon-11 for positron emission tomography (PET) imaging in monkeys [30]. Peak radioactivity was observed in a region rich in TSPO 40 min after tracer injection, with a maximal accumulation in putamen between 12 and 32 min. After the pre-blocked administration of [^11^C]PK 1119519 ([^11^C]1, the rapid uptake in all the examined regions and rapid washout demonstrated the specific binding of [^11^C]31. PET images at 4–100 min post-injection demonstrated a high uptake in the putamen and cerebellum and intermediate levels of accumulation in the cortical regions. Ligand 31 exhibited high-affinity binding in HABs and MABs, which was lower for low-affinity binders even if the authors suggested that further studies in the larger group were needed to establish if differences between binding toward MABs and HABs could be revealed.

**Figure 8:**
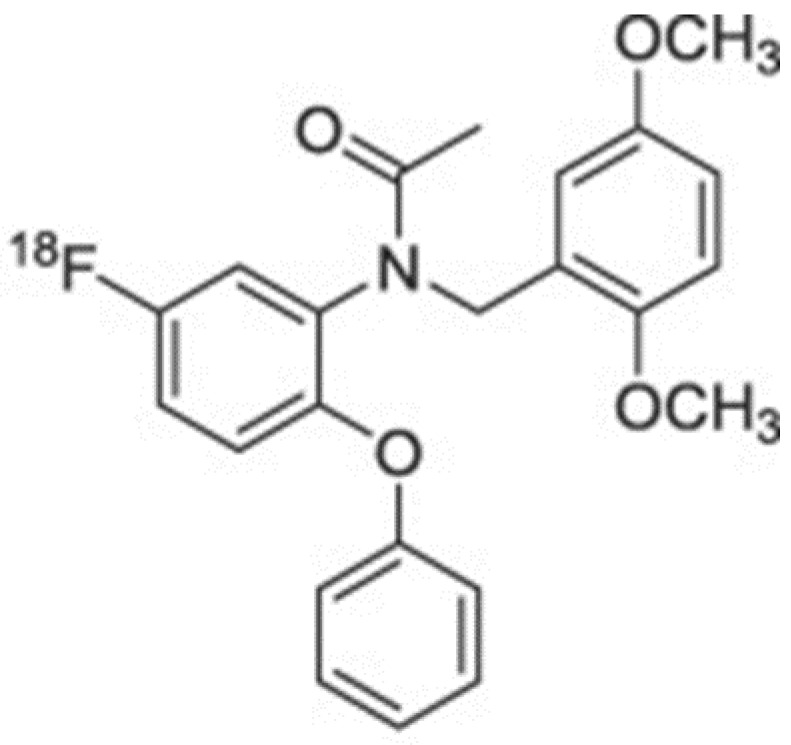
Chemical structure of N-(2,5-Dimethoxybenzyl)-N-(5-fluoro-2-phenoxyphenyl)acetamide (DAA1106) labeled with ¹⁸F belonging to the phenoxyphenylacetamide family.

DAA1106 (Figure 8), a molecule with high binding selectivity for cerebral rat and monkey mitochondria with a weak affinity for CBR, GABA, and Kappa1 receptors, was tested by Kumata et al. [31]. They labeled DAA1106 with fluorine-18 in a previous study in 2007 through the fluorination of diphenyliodonium salt used as a precursor, but, due to the instability of the diphenyl iodonium precursor, the clinical use of the compound was not feasible. This was until the possibility of adding nucleophilic ^18^F into an electron-rich benzene ring, Cu-mediated radiofluorination of pinacol arylboronic ester, or the use of arylstannane precursors with [^18^F]F− and high radiochemical yields were introduced [33,34,35,46]. In their later paper, Kappal et al. ventured into the synthesis of [^18^F]DAA1106 via the [^18^F]fluorination of a spirocyclic iodonium ylide (SCIDY), compound (1), used as a precursor with [^18^F]F−. The SCIDY precursor was demonstrated to be stable at room temperature for 60 days. DAA1106 was stable after radiolabeling with [^18^F] for the duration of at least one PET scan. Biodistribution studies executed on rats al 30 and 60 min after tracer injection showed high uptake in the heart, lungs, spleen, and kidneys and, interestingly, a low uptake in bone, which indicated that no defluorination had occurred thanks to the direct introduction of fluorine-18 in the benzene ring. The polar metabolite generated in rats did not pass the blood–brain barrier or, if penetrated, did not remain in brain tissue due to its hydrophilicity. For the PET study, an ischemic rat brain model was used and demonstrated that [^18^F]DAA1106 had a high binding specificity for TSPO-expressing ischemic areas.

In 2012, in another study by Wadsworth et al., fluorine-18 analogues of DAA1106 were developed [35]. DAA1106 had a high affinity and less lipophilicity with better brain biodistribution. They valued the structure–activity relationships of the analogues by varying their aromatic rings. The reduction of their nitro groups was performed, followed by the reduction of their alanine. Acetylation or fluoroacetylation allowed them to obtain the desired molecule which was tested for affinity screening. Less lipophilicity was obtained by the substitution of phenyl groups with aliphatic groups. The aromatic rings, instead, gave rise to compounds with a higher affinity. The biodistribution of the compounds was studied in naïve Wistar rats. They had a higher whole brain uptake when compared to PK11195 but had a good capacity in discriminating high TSPO-expressing areas and lower ones. In vivo metabolic stability studies stated that compounds **27** and **30** reached the target. In a platelet binding assay however, the question of the affinity of the two binding sites (high affinity, low affinity) of TSPO ligands was not answered.

### 4.2. Studies Conducted on Animals and Humans

Some of the selected studies were performed on animals but also on humans for in vitro examination, in vivo biodistribution, and metabolism studies or post-mortem binding examination.

^11^C-PBR28 was demonstrated to be a valid radiopharmaceutical for the detection of glial response in a study by Donat et al. [9]. A transgenic mouse model of AD was used. The tracer was injected in the tail vein through a cannula and 60 min of dynamic PET scanning was executed. The activity of 370 MBq was also administered to AD patients and then 90 min PET images were acquired. CT scans were obtained for attenuation, scatter correction, and to add morphostructural information to the PET images. To improve the spatial resolution of the PET results, autoradiography with 3H-PBR28 and immunochemistry were executed to provide a tracer density of PBR28 binding sites in mouse brains. During the in vivo characterization of TSPO density through autoradiography, higher microglial representation was associated with higher 3H-PBR28 binding, with major representation in the cortical and hippocampal regions in the AD mouse model.

Arlicot et al. conducted a 90 min dynamic PET scan in seven healthy patients post 245 ± 45 MBq injection of [(^18^)F]DPA-714 [36]. Arterial and venous samples were taken while two volunteers underwent whole-body acquisition 1 h after the tracer’s administration. PET imaging post-processing showed a maximum cerebral uptake in the pons and cerebral radioactive peak within 5 min with a rapid clearance between 5 and 30 min. In whole-body scans, high uptake was seen in the vertebral bodies, gallbladder, heart wall, spleen, intestinal wall, kidneys, and adrenal gland. They also conducted biodistribution studies on mice highlighting similar results.

Starting with the tetracyclic indole-based pharmacophore described in the previously reported by Okubo et al., Wadsworth et al. developed a new tracer, the tricyclic derivative [^18^F]GE180 ([^18^F]5, flutriciclamide) [37]. It demonstrated in rats a high specific binding, a relevant brain uptake with significant uptake and retention in the olfactory bulb, a region rich in TSPO expression, and a good clearance from the region with low TSPO density, that is, the striatum.

Chau et al. tested the higher affinity of the S-enantiomeric tricyclic indole compound, [^18^F]GE-180, a third-generation tracer for the detection of TSPO [19]. In the Wistar rat heart, the S-enantiomer was demonstrated as having a higher affinity with a Ki of 0.87 nM, while the R-GE180 enantiomer had a Ki equal to 3.87 nM. Analogous results were demonstrated in humans (human colonic cell membranes). S-enantiomer radiolabeled with ^18^F had a rapid clearance from blood followed by racemate and R-[^18^F]GE-180. Brain retention in rats was higher in S- radiolabeled enantiomers than R-enantiomers at 10 and 30 min post-injection. S-[^18^F]GE-180 also had a greater lung uptake than the R-enantiomer and racemate and was demonstrated to be stable in vivo without conversion to a distomer.

Nag et al. reported the radiosynthesis of [^18^F]fluorovinpocetine, the fluorinate analogue of [^11^C]vinpocetine [38]. During the autoradiography assay, homogeneous binding between the cortical and subcortical regions was seen in whole hemisphere human brain slices of healthy subjects. Two cynomolgus monkeys underwent a PET scan and a rapid tracer accumulation was observed in the first 4 min with a decrease in around 20 min. [^18^F]fluorovinpocetine had a good brain penetration and similar brain distribution pattern to [^11^C]vinpocetine (high values in the thalamus and striatum, low in the cerebellum), but smaller regional differences.

Damont et al. synthesized a group of pyrazolo[1,5-a]pyrimidines, related to 2, DPA-714 (compound **2** in Figure 3 is represented labeled with ^18^F) to test the in vitro binding affinity of TSPO [39]. Fluroalakyl- and fluoroalakynyl- analogues were created via Sonogashira coupling reactions. In competition experiments against [3H]1 ([3H]-PK11195) in a membrane rat heart, all compounds had a subnanomolar affinity compared to compound **2**, but compound **12** of the fluoroalkyl series and compound **23** from the alkynyl series had the lowest Ki. The specificity of linking was exquisite in all compounds except for **29** and **30**, which showed a modest affinity for central benzodiazepine receptor (CBR). New compounds (**12**, **21**–**24**, and **28**–**30**) have undergone oxidative metabolism investigation in humans, rats, and mouse hepatic microsome assays. In murine microsomes, 90% of biotransformation occurred in 20 min, while in human microsomes, the process was variable in different molecules (31% in 2, 91% in 29). In general, alkynyl derivatives were less metabolized than alkyl compounds. Oxidation did not generate fluoroacetate and so non-brain penetrant species could degrade PET image quality. On the basis of these results, compounds **12** and **23** were chosen for fluorine-18 radiolabeling. Wistar rats underwent PET scans with [^18^F]12 and [^18^F]23 7 days after AMPA-induced brain inflammation in the right striatum. Selective tracer uptake in the right striatum was seen 2 min post tracer injection and maintained for 60 min even though it became slightly lower. A better contrast was obtained with [^18^F]23 due to the rapid washout in the non-lesioned striatum.

### 4.3. Studies Conducted on Humans

In this paragraph, studies conducted on humans are reported, including an in vivo biodistribution evaluation and post-mortem examination. Okello et al. conducted different studies on microglial activation and PET imaging. In their study in 2009, they used the fibrillar amyloid tracer ^11^C-PIB (Pittsburgh compound B) and the peripheral benzodiazepine binding site ligand ^11^C-(R)-PK11195 to explore the possible correlation between microglial activation and Aβ plaque in amnestic mild cognitive impairment (MCI) [40]. A total of 50% of the included 14 subjects with amnestic MCI had increased amyloid deposition but no correlation between the regional C-11-labeled PK-11195 binding and PIB uptake was seen in this group. The authors interpreted this result as due to the multifactorial activation of microglia as a response to amyloid deposition.

^11^C-(R)-PK11195 was used also by Cagnin et al. for Alzheimer’s type dementia (AD), including mild and early forms [41]. The in vivo detection demonstrated high levels of tracer binding in these patients, hinting that the microglial response is an event that could happen in the precocious phase of pathogenesis. In 2004, the same group of researchers demonstrated the presence of higher levels of ^11^C-(R)-PK11195 in people with frontotemporal lobar degeneration, suggesting microglial activation that went hand-in-hand with neuronal loss. Both in AD and frontotemporal dementia, the tracer uptake was independent of the augmented formation of amyloid plaque [41].

Interesting estimations of ([^18^F]5 were conducted in human brains by Feeney et al. [42] and previously, by Zanotti-Fregonara et al. [43], which stated ^18^F-GE180 as an unfavorable tracer for TSPO brain imaging compared with ^11^C-PBR28 because of ^18^F-GE180’s lower brain penetration.

Gulyás et al. [44] performed an in vitro autoradiography on human post-mortem brains of patients who suffered from Alzheimer’s disease by evaluating the binding on TSPO of N-(5-[125I]Iodo-2-phenoxyphenyl)-N-(2,5-dimethoxybenzyl) acetamide ([^125^I]desfluoro-DAA1106) and N-(5-[^125^I]Fluoro-2-phenoxyphenyl)-N-(2-[^125^I]Iodo-5-methoxybenzyl)acetamide ([^125^I]desmethoxy-DAA1106). Their research demonstrated effective binding for both tracers with higher uptake in the hippocampus, temporal and parietal cortex, basal ganglia, and thalamus. Through a comparison with healthy age-matched controls, the tracer had high specificity in microglia-activated areas in the Alzheimer’s brain, confirmed by immunohistochemical examination.

[^18^F]GE180 was also studied in a pharmacokinetics study by Fan et al. in older healthy adults, which stated the two-tissue compartment model was the better model to describe brain kinetics and that 90 min was the ideal scan length for a good assessment [45].

## 5. Conclusions

TSPO represents a hallmark of neuroinflammation. The data from our systematic review demonstrate the huge effort in developing more and more specific tracers. The long half-life of ^18^F (110 min) makes this isotope a preferable choice compared to the shorter life of ^11^C (20 min). Moreover, new chemical synthesis technologies have permitted researchers to improve radiotracer production and allowed research to increase the use of radiolabeling procedures. However, an observed limitation to this research was the huge widespread neuroinflammation involvement that hampers the possibility of detecting a slight inflammation status change in patients. This issue finds a partial solution using the cerebellum as a reference region, as observed in the mentioned studies on ^11^C-PBR28-TSPO. Improvement in the radiosynthesis and detection of molecules with higher TSPO affinity has permitted research to overcome this issue. To cite some results, [^11^C]SSR18057 has a better capacity in discriminating healthy tissue compared to [^11^C](R)-PK11195, while the choice of fluorinated compounds such as ^18^F-DPA-714 has made it possible to obtain a greater binding affinity compared to ^11^C-DPA-713 and ^11^C-PK11195, as demonstrated by Chauveau et al.

Different synthesis strategies have been also exploited to ensure a better stability of the compound: the fluorination of diphenyliodonium salt used as a precursor was demonstrated to not be a feasible approach while the synthesis of [18F]DAA1106 via [18F]fluorination of the SCIDY precursor compound **1** was demonstrated to guarantee a more stable compound for a considerable time. Moreover, [18F]DAA1106 could be used to detect ischemic areas due to its high binding specificity for TSPO. DAA1106 also has a better brain distribution. In particular, the substitution of its phenyl groups with aliphatic groups permits obtaining compounds with less lipophilicity while its aromatic ring, instead, is responsible for higher affinity. Similar consideration could be directed at ^18^F-GE180, which is an unfavorable tracer for TSPO brain imaging for lower brain penetration compared with ^11^C-PBR28.

The introduction of [123I]-CLINME has been interesting in opening the possibility of conducting TSPO-SPECT imaging. The competition studies conducted with PK11195 and Ro 5-4854 have confirmed the specificity of TSPO binding even if thyroid uptake represents a relevant aspect to consider for dosimetry in humans.

Finally, the necessity to consider the presence of distomers or racemic compounds in the synthesis process should be emphasized, which could interfere with the pharmacodynamic and pharmacokinetic effects of the tracer, generating metabolites incapable of overcoming the blood–brain barrier and/or that could increase the noise ratio in PET or SPECT images. The complexity of these subjects was not evaluated in this review and requires further efforts.

## Figures and Tables

**Table 1:** Quality assessment results.

Study	Risk of Bias				Applicability Concerns		
	P	I	R	FT	P	I	R
Donat et al., 2018 [9]	?	✓	✓	✓	✓	✓	✓
Chauveau et al., 2011 [24]	✓	✓	✓	✓	✓	✓	✓
Chauveau et al., 2009 [25]	✓	✓	✓	✓	✓	✓	✓
Vignal et al., 2018 [26]	✓	✓	✓	✓	✓	✓	✓
Solingapuram et al., 2015 [27]	✓	✓	✓	✓	✓	✓	✓
Mattner et al., 2015 [28]	✓	✓	✓	✓	✓	✓	✓
Tran et al., 2019 [29]	✓	✓	✓	✓	✓	✓	✓
Qiao et al., 2019 [16]	✓	✓	✓	✓	✓	✓	✓
Pike et al., 2011 [30]	✓	✓	✓	✓	✓	✓	✓
Kumata et al., 2018 [31]	✓	✓	✓	✓	✓	✓	✓
Zhang et al., 2007	✓	✓	✓	✓	✓	✓	✓
Tredwell et al., 2016 [32]	✓	✓	✓	✓	✓	✓	✓
Zischler et al., 2016 [33]	✓	✓	✓	✓	✓	✓	✓
Zischler et al., 2017 [34]	✓	✓	✓	✓	✓	✓	✓
Wadsworth et al., 2012 [35]	✓	✓	✓	✓	✓	✓	✓
Arlicot et al., 2011 [36]	✓	✓	✓	✓	✓	✓	✓
Wadsworth et al., 2012 [37]	✓	✓	✓	✓	✓	✓	✓
Chau et al., 2015 [19]	✓	✓	✓	✓	✓	✓	✓
Nag et al., 2019 [38]	✓	✓	✓	✓	✓	✓	✓
Damont et al., 2015 [39]	✓	✓	✓	✓	✓	✓	✓
Okello et al., 2009 [40]	?	✓	✗	?	✓	✓	✓
Cagnin et al., 2001 [41]	✓	✓	✓	✓	✓	✓	✓
Cagnin et al., 2004	✓	✓	✓	✓	✓	✓	✓
Feneey et al., 2016 [42]	✓	✓	✓	✓	✓	✓	✓
Zanotti-Fregonara et al., 2018 [43]	✓	✓	✓	✓	✓	✓	✓
Gulyás et al., 2009 [44]	✓	✓	✓	✓	✓	✓	✓
Fan et al., 2016 [45]	✓	✓	✓	✓	✓	✓	✓
Preshlock et al., 2016 [46]	✓	✓	✓	✓	✓	✓	✓

P = patient selection; I = index test; R = reference standard; FT = flow and timing. ✓ indicates low risk; ✗ indicates high risk; ? indicates unclear risk.

**Table 2:** QUADAS-2—Number of studies at low, high, or unclear risk of bias and concerns regarding applicability.

	Risk of Bias				Concerns Regarding Applicability		
	Patient Selection	Index Test	Reference Standard	Flow and Timing	Patient Selection	Index Test	Reference Standard
Low	26	28	27	27	28	28	28
High	0	0	1	0	0	0	0
Unclear	2	0	0	1	0	0	0
Total	28	28	28	28	28	28	28

## Data Availability

Not applicable.
